# Discrimination of *Salvia miltiorrhiza* Bunge from Different Geographical Locations Employing High-Performance Liquid Chromatography, Near-Infrared Fingerprinting Combined with Chemometrics

**DOI:** 10.1155/2020/8367619

**Published:** 2020-02-10

**Authors:** Jiao Wang, Yichun Sun, Zhan Li, Wei Li, Yuanyuan Pang, Jiayu Li, Qing Wu

**Affiliations:** ^1^Guizhou Key Laboratory for Information System of Mountainous Areas and Protection of Ecological Environment, Guizhou Normal University, 116 Baoshan North Rd, Guiyang 550001, Guizhou, China; ^2^Tong Ji Tang (Guizhou) Pharmaceutical Co., Ltd., Guiyang, Guizhou 550018, China; ^3^Guiyang University of Chinese Medicine, Guiyang, Guizhou, 550018, China; ^4^Guiyang No. 3 Experimental High School, Guiyang, Guizhou 550018, China

## Abstract

To evaluate the quality of *Salvia miltiorrhiza* Bunge, high-performance liquid chromatography-diode array detector (HPLC/UV-PAD), near infrared (NIR) spectroscopy, and chemometrics were used to discriminate nine components of samples from four different geographical locations. HPLC was performed with a C18 (5 *μ*m, 4.6 mm × 250 mm) column and 0.1% formic acid aqueous solution-acetonitrile with a gradient elution system. Orthogonal partial least squares discriminant analysis was used to identify the amounts of salvianolic acid B. NIR was used to distinguish rapidly *S. miltiorrhiza* Bunge samples from different geographical locations. In this assay, discriminant analysis was performed, and the accuracy was found to be 100%. The combination of these two methods can be used to quickly and accurately identify *S. miltiorrhiza* Bunge from different geographical locations.

## 1. Introduction

For the purpose of prevention and treatment of diseases in humans and animals, medicinal plants are applied globally [1, 2]. As reported by the World Health Organization (WHO), about 80% of the world's population from developing and underdeveloped countries use plant-derived medicines for health care [3]. Clinical studies demonstrated that many medicinal plants can efficiently regulate the human immune system and inflammation [4, 5]. In 2002, The Food and Drug Administration (FDA) approved 39 new drugs including two botanical drugs (oral and topical) [6].

The chemical compositions of traditional Chinese medicines (TCMs) are complex; the quality of TCM cannot be comprehensively and effectively controlled only by one or several [7–9]. While attracting increasing attention, chemical fingerprints have been approved by the WHO and FDA as a method for medicine identification and quality assessment. Compared with conventional analytical approaches, fingerprint techniques can be combined for the holistic characterization of a test sample in a complex system [10, 11]. Numerous chemical fingerprint methods for discrimination and quality evaluation of TCM have been introduced, including near-infrared (NIR) spectroscopy, Fourier-transform infrared (FTIR) spectroscopy, high-performance liquid chromatography (HPLC), and electronic nose and electronic tongue [12–18].

As a medicinal plant, *Salvia (S.) miltiorrhiza* Bunge is very popular in China. Dry roots and rhizomes of *S. miltiorrhiza* are called Dan Shen in Chinese pharmacopoeia (Dan Sam in Korean and Tan Shen in Japan) [19]. In *S. miltiorrhiza*, with regard to the chemical properties and structural characteristics, the principal bioactive components fall under two categories: diterpenoid quinones (e.g. tanshinone I, tanshinone IIA, and cryptotanshinone) and hydrophilic phenolic acids (e.g. salvianolic acid A and salvianolic acid B) [20–23]. As one of the most versatile Chinese herbal drugs, Dan Shen has been applied for the treatment of various diseases, including cerebrovascular and coronary heart diseases [24–27]. Chinese medicinal materials are directly affected by the changes in their growing environment and processing and storage conditions. The quality and curative effect of traditional Chinese medicine are directly related to the type and content of active ingredients [28–31].

In the current study, based on the techniques of ultraviolet (UV) spectroscopy and NIR with principal component analysis (PCA), a practical method was developed for the discrimination of *Salvia miltiorrhiza* Bunge from various geographical origins. The method was founded to analyze multiple active ingredients of the specimens. The spectroscopic data were analyzed by methods for chemometric pattern recognition, such as hierarchical cluster analysis (HCA) and partial least squares discrimination analysis (PLS-DA). This method provides more information for rapidly and accurately discriminating *Salvia miltiorrhiza* Bunge.

## 2. Materials and Methods

### 2.1. Samples

**Figure 1:**
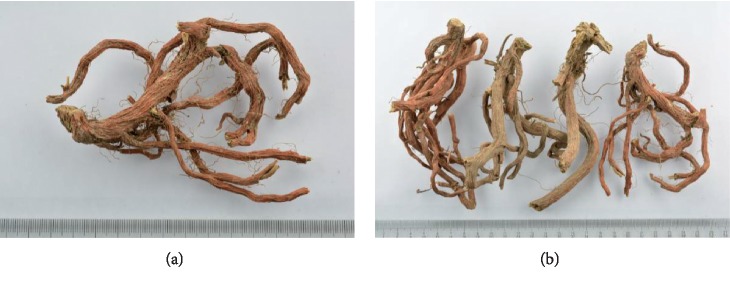
(a) *Salvia miltiorrhiza* Bunge voucher specimen; (b) *Salvia miltiorrhiza* Bunge from four different geographic locations: Sichuan, Shandong, Henan, and Anhui provinces in China, respectively.

The sampling was carried out in four geographical origins, and 120 raw *Salvia miltiorrhiza* Bunge samples (ca. 200–300 g each) were collected in total: 35 samples from Zhongjiang city, 30 samples from Fangcheng city, 25 samples from Juxian city, and 30 samples from Quanjiao city, as shown in Figure 1. The four different origins are Si Chuan, Henan, Anhui, and Shandong provinces in China, respectively. Samples were acquired from the four provinces during the harvest seasons in 2017 and 2018. All samples were collected and authenticated by Professor Wei Shenhua from Guiyang University of Chinese Medicine.

### 2.2. Reagents

Standards of protocatechuic aldehyde (PTAL), protocatechuic acid (PTA), salvianolic acid B (SAB), caffeic acid (CA), and cryptotanshinone (CTS) were purchased from National Institutes for Food and Drug Control. Dihydrotanshinone I (DI), rosmarinic acid (RA), tanshinone II A (TSIIA), and tanshinone I (TSI) were provided by Sichuan Weikeqi Biological Technology Co., Ltd, (Chengdu, China). Their purities were determined ≥98% as by HPLC based on a peak area normalization method. Formic acid and acetonitrile of HPLC grade were purchased from Fisher Scientific Co. (CITY, NJ, USA). Purified water was purchased from GuiYang Wahaha Group. All other chemicals and reagents were purchased from the Sinopharm Chemical Reagent Co., Ltd. (Beijing, China) and were of analytical grade.

### 2.3. HPLC Analysis

#### 2.3.1. Preparation of Standard Solution

For each standard, one milligram (mg) was precisely weighed and dissolved in 5 mL methanol to produce standard solutions. The concentrations were 0.88 mg/mL protocatechuic acid, 0.68 mg/mL protocatechuic aldehyde, 0.61 mg/mL caffeic acid, 1.88 mg/mL rosmarinic acid, 2.24 mg/mL salvianolic acid B, 0.65 mg/mL dihydrotanshinone I, 0.67 mg/mL cryptotanshinone, 0.46 mg/mL tanshinone I, and 0.43 mg/mL tanshinone IIA. Then, the standard solution was mixed to make a new solution containing 2.64 *μ*g/mL protocatechuic acid, 1.36 *μ*g/mL protocatechualdehyde, 3.66 *μ*g/ml caffeic acid, 37.6 *μ*g/ml rosmarinic acid, 0.896 mg/ml salvianolic acid B, 6.5 *μ*g/ml dihydrotanshinone I, 13.4 *μ*g/ml cryptotanshinone, 7.36 *μ*g/ml tanshinone I, and 6.45 *μ*g/ml tanshinone IIA. The new solution corresponding to each standard solution was prepared and then stored at 4°C in a dark glass bottle. The working standard solutions were prepared freshly via diluting certain volumes of the prepared stock solution with methanol.

#### 2.3.2. Preparation of Sample Solutions for HPLC Analysis

All *S. miltiorrhiza* samples were crushed into powder and sifted through 60 mesh screens. As for the preparation of sample solutions for further analysis, roughly 1 g of the *S. miltiorrhiza* sample was weighed and then extracted by 50 mL of 50% methanol. The extraction was carried out in ultrasound for 60 min. The sample was weighed in another 50% methanol, which was added to ensure the conservation of mass. Before HPLC analysis, a 0.45 *μ*m organic phase filtration membrane was applied to filter the extraction solution.

#### 2.3.3. Chromatographic Conditions

Chromatographic separation and determination of the extraction solutions were conducted employing an Agilent HPLC system (Agilent, Palo Alto, CA, USA) equipped with a quaternary pump, an online degasser, an autosampler, a column temperature controller, and a diode-array detector (Agilent, USA). The Diamonsil C18 column (5 *μ*m, 4.6 mm × 250 mm) was used for all analyses. The mobile phase comprised 0.1% aqueous formic acid (A) and acetonitrile (B), and the following gradients were used: 0–10 min:10–20% B, 10–17 min:20% B, 17–45 min: 20–33% B, and 45–90 min: 33–100% B. A volume of 10 *μ*L was set as the injection volume and the following detecting wavelengths of the UV detector were used: 0–14 min: 270 nm; 14–33 min: 280 nm; 33–40 min: 270 nm; 40–65 min: 280 nm; and 65–90 min: 270 nm. The flow rate and column temperature were set as 1.0 mL/min and 35°C, respectively.

#### 2.3.4. Validation of Quantitative Analysis Method

The quantitative analysis methods for protocatechuic acid, protocatechualdehyde, caffeic acid, salvianolic acid B, rosmarinic acid, dihydrotanshinone I, tanshinone I, cryptotanshinone, and tanshinone IIA were validated via parameters such as precision, accuracy, and linearity.

*(1) Linearity*. For each compound, six concentrations were used to construct the corresponding calibration curve. The plotting of the calibration graph was based on the linear regression analysis of the integrated peak areas versus concentrations of the standard. Appropriate concentrations were obtained by diluting the standard solution with methanol.

*(2) Precision, Stability, and Recovery*. In order to determine the precision for plant material samples containing the nine analytes, and the relative standard deviation (RSD) value calculation were carried out for the integration area.

The intraday variability of one sample was determined by six times in 24 h (0, 2, 4, 8, 12, and 24 h), while the determination of the daily precision was performed on four consecutive days. Nine independent sample solutions were used to determine the precision of the assay method. For accuracy evaluation, a standard addition method was used to perform the recovery test. Half the plant material (ca. 0.50 g) was used for the test sample preparation. Three volumes of standard solution containing the three analytes were spiked into the sample solution. Therefore, the concentration of each analyte in the standard solution was 50%, 100%, and 150% of that in the half mass sample solution, respectively. Afterwards, the extraction of the sample was performed via the procedure as demonstrated in Section 2.3.2 for HPLC analysis.

#### 2.3.5. Validation of HPLC Fingerprint Analysis Method

The intraday variability of one sample was determined by six times in 24 h (0, 2, 4, 8, 12, and 24 h), while the determination of daily precision was performed on three consecutive days, which was based on the relative peak area RSD values and relative retention time (in comparison to salvianolic acid B) of all integrated peaks. An assay was repeated on six independent sample solutions.

### 2.4. NIR Analysis

#### 2.4.1. Instrumental Conditions

NIR spectra were recorded employing an AntarisII Fourier Transform NIR spectrometer (Thermo Fisher Company, USA). The measurement was conducted with an integrating sphere in reflectance mode, at room temperature (25 ± 2°C) with a humidity of 40%∼60%.

#### 2.4.2. Sample Spectrum Collection

The NIR absorption intensity-*Salvia miltiorrhiza* Bunge size (i.e., intact sample, 24 mesh, 50 mesh, 80 mesh, and 120 mesh) relationship was evaluated, and a positive phase proportion was founded, and the absorption intensity of intact *Salvia miltiorrhiza* Bunge was the weakest while the powdered *Salvia miltiorrhiza* Bunge (120 mesh) was the strongest, without any relationship obtained at other wavelengths. Approximately 2 g of powdered *Salvia miltiorrhiza* Bunge (120 mesh) was placed on a select integral ball diffused reflection optical platform in a cylindrical quartz sample holder. The scanning range was 12,500 to 4000 cm^−1^. The spectra were collected 3 times for each sample, 32 times of scanning, resolution factor of 4 cm^−1^, and the average of the 3 acquired spectra was used as the spectrum of the sample.

### 2.5. Statistical Data Analysis

Both principal component analysis (PCA) and orthogonal partial least squares discriminant analysis (OPLS-DA) were completed. SIMCA-P Version 14.0 software (Umetrics AB, Umea, Sweden) and SPSS (IBM SPSS Statistics version 24.0, Armonk, NY, USA) were used to analyze the HPLC data.

Two sets of *Salvia miltiorrhiza* Bunge samples were sorted randomly: the model set containing 3/4 of the samples for the statistical model construction, and the external validation set containing the remaining 1/4 for model validation.

## 3. Results and Discussion

### 3.1. Optimization of Sample Extraction

**Figure 2:**
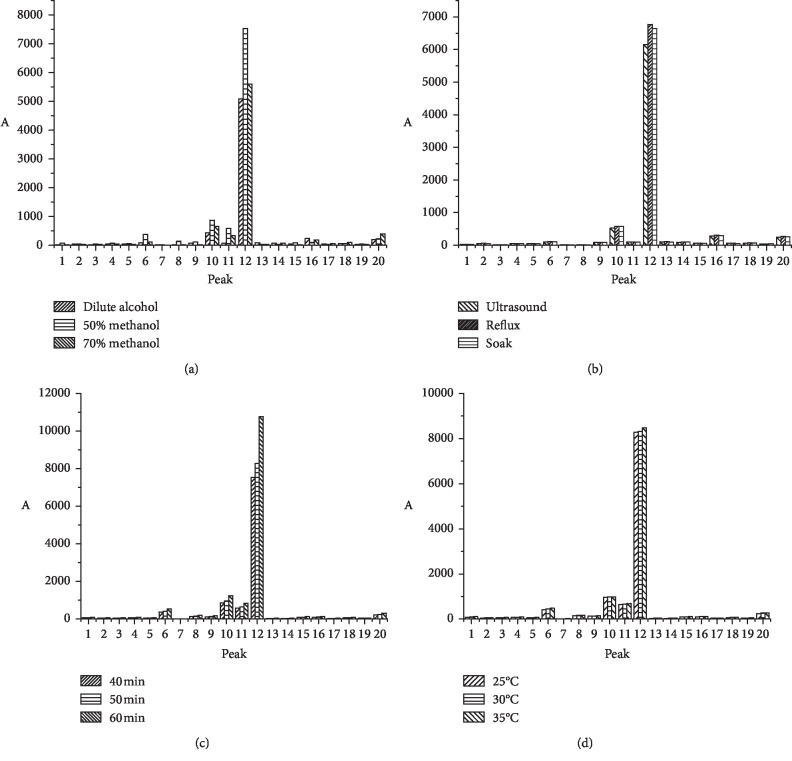
The peak area of 20 common peaks were compared under different extraction solvents (a), methods (b), time (c), and temperature (d).

To increase the amount of active ingredients acquired and improve sample fingerprints, the orthogonal experiment was applied to optimize variables involved in the extraction process, e.g., the method, solvent, and time, as shown in Table 1. After comparing the peak area of 20 common peaks, the results showed the efficiency of various extraction solvents, i.e., 50% methanol, 70% methanol, and dilute alcohol. As shown in Figure 2(a), 50% methanol exhibited higher extraction efficiency with better fingerprints compared with the others. Then, different methods, including ultrasound-assisted extraction, Soxhlet extraction, and room temperature soaking, were compared. No significant difference was observed among the three methods (Figure 2(b)). However, in terms of time efficiency and ease of operation, ultrasound-assisted extraction was applied for further experiments. The influence of ultrasonication time (40, 50, and 60 min) on sample extraction in 50% methanol was then investigated. The results showed that extraction was effective between 50 and 60 min in Figure 2(c). Finally, single factor was used to investigate different extracted temperatures gradient at 25, 30, and 35°C. The results indicated that extraction was effective at 35°C, as shown in Figure 2(d). The optimal extraction conditions for *S. miltiorrhiza* Bge are summarized and presented in Section 2.3.2.

### 3.2. Optimization of Chromatographic Conditions

**Figure 3:**
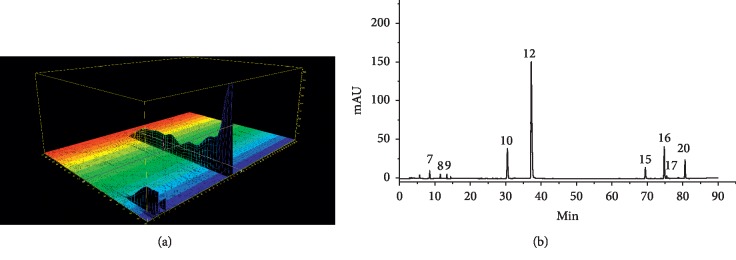
(a) DAD full scan of reference substances. (b) Chromatogram of reference substances: 7, protocatechuic acid; 8, protocatechualdehyde; 9, caffeic acid; 10, rosmarinic acid; 12, salvianolic acid B; 15, dihydrotanshinone I; 16, cryptotanshinone; 17, tanshinone I; and 20, tanshinone II A.

Based on our preliminary procedure experiments, combinations of different mobile phase solvents (i.e. acetonitrile and methanol-water with different modifiers, including formic acid and phosphoric acid solution) were applied. The associated results of different flow rates (0.8, 1.0, and 1.2 mL/min) of the mobile phase, wavelengths (270, 286, and 360 nm), and column temperatures (30, 35, and 40°C) were investigated and compared. The determination of optimized chromatographic conditions was based on the total analysis time, and the number and resolution of peaks. The binary mixtures of acetonitrile and 0.1% aqueous formic acid exhibited low pressure and greater baseline stability. The maximum absorption wavelength of each component is different. In order to ensure that the maximum extent of these nine components were tested, as shown in (Figure 3(a)), the ultraviolet spectra of the components at 200–400 nm were compared. The final determination of detection wavelengths were 0–14 min: 270 nm; 14–33 min: 280 nm; 33–40 min: 270 nm; 40–65 min: 280 nm; and 65–90 min: 270 nm. Maintaining the column temperature at 35°C, a flow rate of 1.0 mL/min provided acceptable peak parameters with good resolution within 90 min (Figure 3(b)). Under the determined optimal conditions, adequate absorption was achieved for most components without any interference.

### 3.3. Validation of Quantitative Analysis Method

The validation of the quantitative analysis method was performed in regard to the linearity, precision, stability, repeatability, and recovery tests. As listed in Table 2, the calculated linear regression analysis results are compared. Over the concentration range tested, a good linearity (correlation coefficient *R* > 0.999) was obtained for all the standard compounds (Table 2). The analyzing of standard solutions enabled the determination of instrument precisions. The stabilities of the nine compounds were tested six times (0, 8, 16, 24, 48, and 96 h). The result (Table 3) indicated a precision variation of below 2.63% for all the analytes that were found to be stable during the testing. The RSD of the nine compounds' stability and repeatability was below 2.62 and 2.35%, respectively (Table 3). A standard spiking test was applied for recovery validation. The method was confirmed to be precise and accurate by an average recovery value of between 91.21 and 99.21% for the marker compounds.

### 3.4. Validation of HPLC Fingerprint Analytical Method

The validation of HPLC fingerprint analytical method was performed in regard to precision, reproducibility, and stability. Since peak 12 presented good shape with stable peak area, and was observed in all chromatograms, and furthermore, located in the middle, the corresponding compound, SAB, was selected as the reference substance for the calculation of common peak RRT and RPA in all samples. For the reproducibility, precision, and stability tests (Table 4), the RSD of RRT and RMA was 0–2.88% and 0–2.98%, respectively, indicating the suitability of the developed HPLC method for *S. miltiorrhiza* fingerprint analysis.

### 3.5. Chromatographic Peak Identification

**Figure 4:**
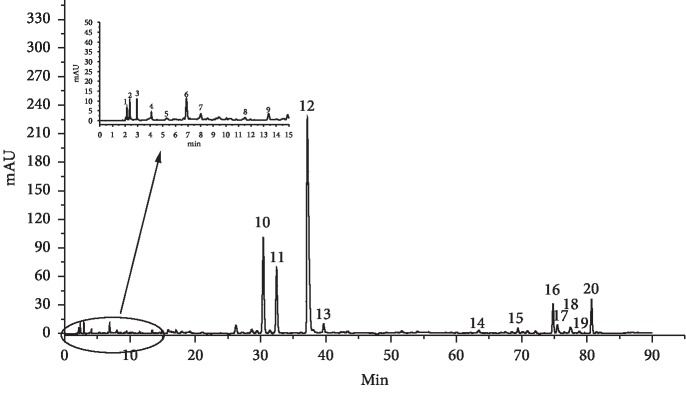
Chromatogram of *S. miltiorrhiza*.

**Figure 5:**
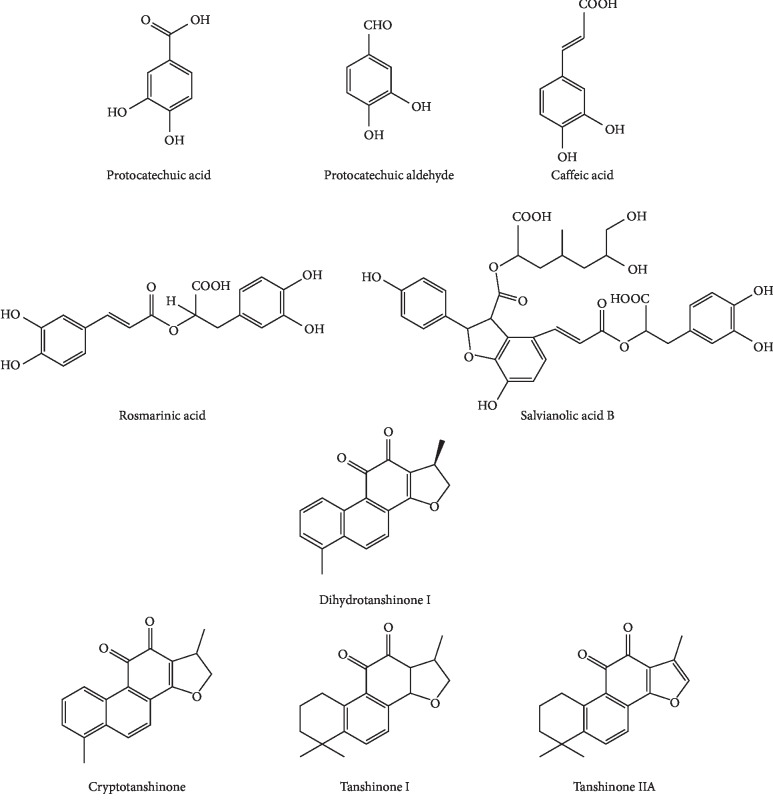
Chemical structures of the nine active compounds identified in *Salvia miltiorrhiza* Bunge.

The *S. miltiorrhiza* sample solutions were prepared following the previously described procedure. The chromatograms of the samples were recorded (Figure 4) to the *S. miltiorrhiza*. Samples' fingerprints were under the HPLC conditions. Peaks with reasonable heights and good resolution in the chromatograms were labelled as “common peaks” to express the characteristics of *S. miltiorrhiza*. In each batch of samples, 20 common peaks were labelled with reasonable resolutions and satisfactory peak signals, displaying an appearance ratio of 90%. As indicated in Figures 3(b) and 4, by matching retention time (RT) with the respective reference compounds, nine common peaks were identified. There are nine active compounds of chemical structures in *Salvia miltiorrhiza* Bunge as shown in Figure 5. Peaks 7–9, 10, 12, 15, 16, 17, and 20 were PTA, PTAL, CA, RA, SAB, DI, CTS, TS I, and TS II A, respectively. The nine bioactive components identified in *S. miltiorrhiza* fall into two categories: diterpenoid quinones (e.g. tanshinone I, dihydrotanshinone I, cryptotanshinone, and tanshinone II A) and hydrophilic phenolic acids (such as protocatechuic acid, caffeic acid, protocatechuic aldehyde, salvianolic acid B, and rosmarinic acid). These active components identified in *S. miltiorrhiza*, i.e., diterpenoid quinones and hydrophilic phenolic acids, possess strong antioxidant, anticancer, antimicrobial, and cardioprotective functions.

### 3.6. Chemical Comparison of Geographical Differences in *S. miltiorrhiza* Based on an Analysis of Multiple Constituents

#### 3.6.1. Cluster Analysis

**Figure 6:**
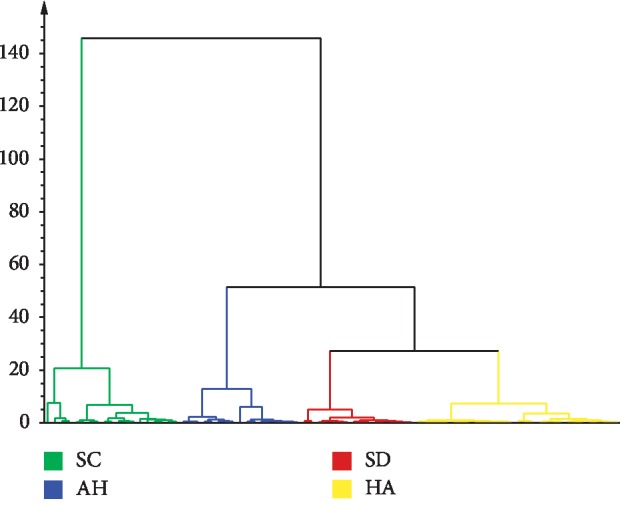
The clustering analysis of 120 batch *S. miltiorrhiza* samples.

Clustering analysis was used to directly compare the properties of samples in a multidimensional space and classify the samples with similar properties into one class, so that the samples from the same class have a high degree of phase similarity. The peak of the 120 batches of extracts from one area was one variable. SIMCA-P Version 14.0 software was used. The clustering analysis results of *S. miltiorrhiza* are shown in Figure 6. The medicinal materials can be divided into three categories and clustering of *S. miltiorrhiza* in different years. Different batches from Henan and Shandong were divided into one category, followed by Anhui and then Sichuan.

#### 3.6.2. Principal Component Analysis

To evaluate the extract to distinguish different geographical *S. miltiorrhiza* locations, SIMCA-P Version 14.0 software was used for PCA scores evaluation.

**Figure 7:**
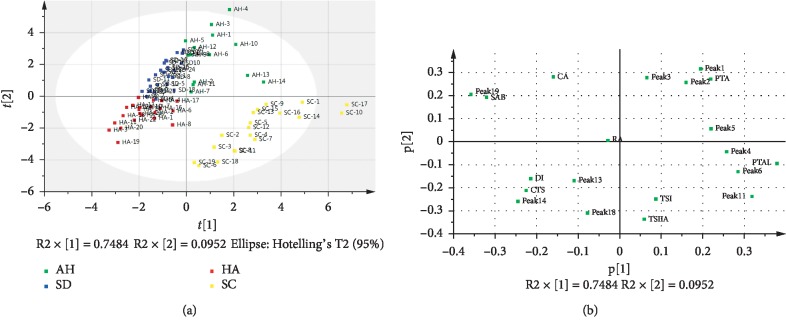
(a) PCA score and (b) loading plots of *S. miltiorrhiza*. HA is Henan; AH is Anhui; SD is Shandong; SC is Sichuan.

HPLC fingerprinting was used for principal component analysis with four principal components. On the basis of eigenvalues greater than one, the first two PCs (PC1 and PC2) were found to explain 74.84 and 9.52% of the variation. The loading plot is shown in Figure 7. According to their loadings, PC1 had good correlation with peaks 13, 14, 18, and RA, DI, TSI, CTS, and TSIIA. PC2 had good correlation with peaks CA, 19, and SAB, which indicates that these compounds may contribute more to the classification of the samples than other.

#### 3.6.3. OPLS-DA Analysis

**Figure 8:**
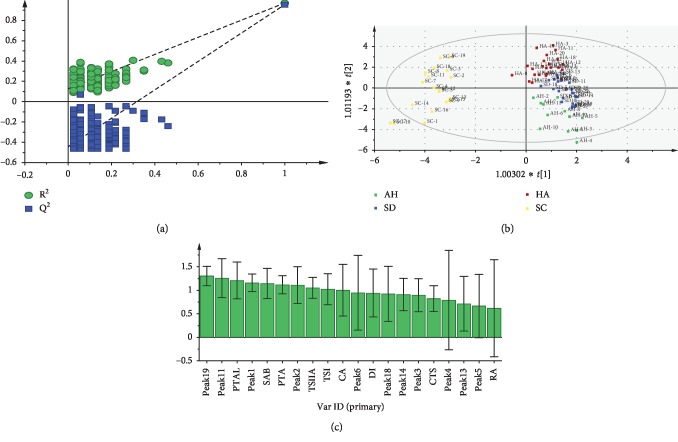
Statistical analyses of samples based on HPLC data. (a) A presentation of chance permutation at 200 times used for the discrimination between the two groups [*R*^2^ = (0.0, 0.104), *Q*^2^ = (0.0, −0.449)]; (b) OPLS-DA score plot of saffron samples from four different geographical locations [*R*^2^*Y* (cum) = 0.835 and *Q*^2^*Y* (cum) = 0.811]; (c) VIP plot for samples based on PLS-DA.

The selected optimal number of latent variables was two for building a PLS-DA model. The values of R2X, R2Y, and Q2 were 0.908, 0.835, and 0.811, respectively, indicating a better fitting and predictive ability of this model in data processing. Subsequently, the model was validated by a permutation test and, according to 200 permutations, the vertical intercept values of R2 and Q2 were 0.104 and −0.449, respectively (Figure 8(a)). This indicated the absence of the overfitting problem for the established model and improved the forecasting accuracy effectively. Based on the two latent variables' 2D score plot (Figure 8(b)), the samples tested were classified into four categories excellently, being consistent with HCA and PCA results. In order to determine the significance of *X* variables on sample discrimination, the VIP profile was constructed (Figure 8(c)). The VIP value was determined to be greater than 1 for many variables, suggesting the great importance of these *X* variables for distinguishing between Danshen samples. Combining the VIP values of each variable, the different compounds from the four geographical samples were determined as chromatographic peaks 11, 19, 1, 2, and 6, and coffee acid, salvianolic acid B, and protocatechuic aldehyde.

### 3.7. NIR Analysis

**Figure 9:**
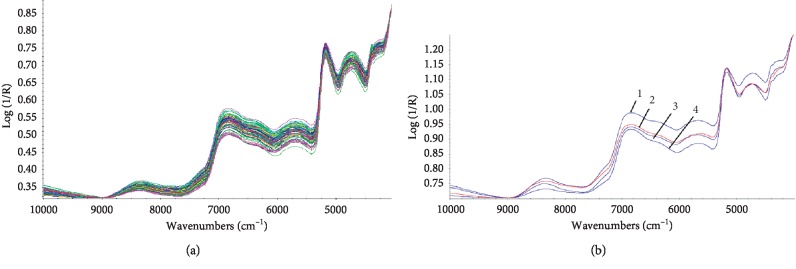
(a) Raw spectra and (b) the average raw spectra and average preprocessed spectra. 1: Sichuan, 2: Shandong, 3: Hena, and 4: Anhui.

The NIR spectrogram of the *Salvia miltiorrhiza* Bunge root sample was input into TQ Analyst. The discriminant analysis provided by the TQ software adopts principal component analysis combined with the Mahalanobis distance. The performance index (PI) of the model is the indicator, and the optimized spectral is normal and constant. The raw spectra (Figure 9(a)) and average raw spectra (Figure 9(b)) of samples from the four different geographical origins were recorded, respectively.

In order to achieve reliability, accuracy, and stability of the model, spectral preprocessing is commonly conducted before model calibration. Several spectral preprocessing algorithms, such as multiplicative scatter correction (MSC), standard normal variate (SNV), first derivative (FD) and second derivative (SD), Norris derivative (ND), and Savitzky-Golay (SG) smoothing, were carried out employing a TQ Analyst software for optimizing the calibration.

The performance index of the model constructed using various spectrum pretreatments is indicated in Table 5. After comparison, we selected the MSC + SD + S − G combination as a pretreatment method.

**Figure 10:**
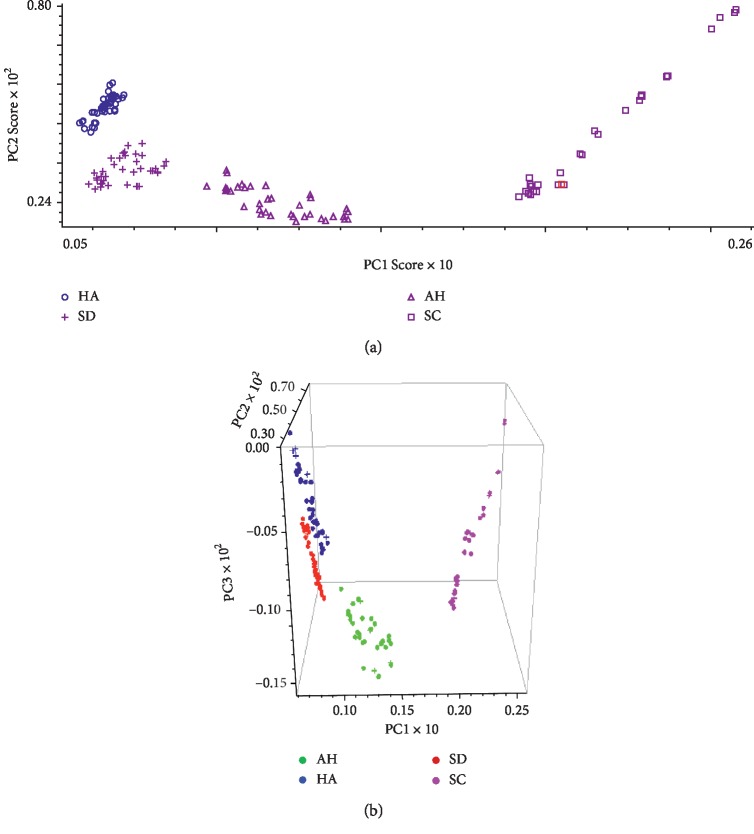
(a) DA scores plot of *S. miltiorrhiza* samples from different geographical origins from four different provinces. (b) Score plot based on the first 10 principal components for the calibration set (dots) and validation set samples (crosses). The sample spectra were pretreated with MSC, SD, and SG.

Figure 10 illustrates the results of the geographical origin discrimination. Based on PC 1–3, i.e., the first three principal components, the score plot corresponding to the three-dimensional (3D; Figure 10(b)) principal component space was obtained. With the cumulative variance contribution rate reaching 95.86% for PCs 1–3, the 3D space can be applied to roughly explain the full spectra. Figure 10 shows a clear boundary between He Nan, Shan Dong, An Hui and Si Chuan classes, which implies the feasibility of discriminating *S. miltiorrhiza* samples according to geographical origin. The He Nan class is similar to the Shan Dong class and An Hui is distinguishable from Si Chuan classes.

Due to the chemical composition discrepancies of samples, caused by different climate and soil conditions, in different geographical origins, the sample spectra may also vary depending on geographical origin. The average distances of samples within each class to different class centers are listed in Table 6. The samples within each class are found to be closer to the corresponding class center (i.e. same class) than to the other class centers. The similarity or dissimilarity among classes is in fundamental accordance with results illustrated in Figure 10. 30 samples were successfully classified in the validation set using the established model. With an accuracy of 100%, each sample was discriminated correctly in terms of geographical origin.

The 120 batches of *Salvia miltiorrhiza* Bunge from different geographical locations were easily able to be classified into Southwest region or Central Plains areas. Samples were classified into four classes according to different provinces. The NIR spectrum contains a large amount of information about the medicinal materials. Environmental differences between Central Plains and Southwest China lead to these differences in the quality of medicinal materials. Thus, the NIR method is feasible for distinguishing samples from different geographical locations.

## 4. Conclusions

In this study, we were able to determine the geographical discrimination of *S. miltiorrhiza*. Two methods were applied for the determination, with each showing different advantages.

An integrated approach combining chemometrics and HPLC was firstly employed to distinguish Central Plains and Southwest China *Salvia miltiorrhiza* Bunge. We found that different geographical samples could be well separated via PCA and OPLS-DA, according to the corresponding HPLC fingerprint data. The volatile ingredient variations were tested. We found that protocatechuic acid, protocatechuic aldehyde, salvianolic acid B, caffeic acid, cryptotanshinone, rosmarinic acid, dihydrotanshinone I, tanshinone I, and tanshinone II A contents were different for different geographical samples. Salvianolic acid B was determined to be the discriminating variable. Subsequently, an integrated approach combining chemometrics with NIR was employed to discriminate the sample origins quickly and nondestructively. The NIR model should be further developed, and NIR discrimination is faster and more accurate than traditional methods.

In this study, the chromatographic fingerprint method was used to comprehensively evaluate the traditional Chinese medicine peculiarity from a macroscopic point of view. We used HPLC fingerprints to compare the peculiarity of different origins of *S. miltiorrhiza*. Near-infrared spectroscopy (NIRS) is a fast and nondestructive green analysis technology that can be used for quality evaluation of Chinese herbal medicines as a whole. The process uses a simple sample, and no reagents are required. It has been widely used in the quality control and identification of Chinese herbal medicines. In this paper, the components of different geographical locations of *S. miltiorrhiza* were evaluated and analyzed by combining the two techniques.

## Figures and Tables

**Table 1:** Orthogonal experiment for *S. miltiorrhiza* extraction.

The serial number	Solvent	Time (min)	Method
1	50% methanol	40	Ultrasound-assisted extraction
2	50% methanol	50	Soxhlet extraction
3	50% methanol	60	Soak at room temperature
4	Dilute alcohol	40	Soxhlet extraction
5	Dilute alcohol	50	Soak at room temperature
6	Dilute alcohol	60	Ultrasound-assisted extraction
7	70% methanol	40	Soak at room temperature
8	70% methanol	50	Ultrasound-assisted extraction
9	70% methanol	60	Soxhlet extraction

**Table 2:** Calibration curves data of nine bioactive compounds in samples.

Analytes	Calibration curves	*R* ^2^	Linear range (*μ*g/mL)
PTA	*y* = 11.053*x* + 1.2873	0.9997	0.528–6.60
PTAL	*y* = 7.327*x* + 0.2632	0.9993	0.272–3.40
CA	*y* = 4.724*x* + 0.1380	0.9997	0.732–9.15
RA	*y* = 362.1*x* − 11.843	0.9996	7.52–94.0
SAB	*y* = 24.02*x* + 0.3742	0.9998	0.179–2.24
DI	*y* = 26.55*x* − 1.2230	0.9995	1.30–16.2
CTS	*y* = 6.866*x* + 0.2087	0.9996	2.68–33.5
TSI	*y* = 26.765*x* − 0.3373	0.9991	1.47–18.4
TSIIA	*y* = 72.333*x* + 2.7490	0.9994	1.29–16.12

**Table 3:** Precision, stability, repeatability, and recoveries of the nine bioactive compounds' relative peak area in samples (*n* = 6).

Analytes	Precision (relative standard deviation, RSD, %)		Repeatability (RSD, %)	Robustness (RSD, %)	Accuracy (*n* = 6)	
	Intraday (*n* = 6)	Interday (*n* = 6)			Mean	RSD%
PTA	1.92	2.12	1.32	1.54	91.21	2.71
PTAL	1.15	1.23	0.86	1.76	93.14	2.13
CA	0.85	1.01	1.14	2.62	94.01	1.94
RA	2.12	2.21	1.53	2.38	96.52	1.62
SAB	1.57	1.62	2.09	1.81	97.68	2.32
DI	2.52	2.49	2.35	0.97	99.05	1.89
CTS	0.89	1.02	1.91	1.27	98.50	2.98
TSI	2.71	2.68	1.57	0.68	99.21	1.45
TSIIA	2.63	2.71	1.75	2.11	94.67	1.22

**Table 4:** Analytical results of precision, stability, and reproducibility tests of 20 characteristic common peaks in *S. miltiorrhiza* (*n* = 6).

Peak	RSD of RRT (%)			RSD of RPA (%)		
no.	Precision	Stability	Reproducibility	Precision	Stability	Reproducibility
1	1.94	1.12	1.46	2.84	2.63	1.75
2	2.83	2.46	0.65	2.33	1.23	2.31
3	0.95	0.87	0.66	1.98	0.96	0.56
4	0.56	1.88	0.34	2.13	2.67	1.63
5	0.21	2.85	0.20	0.67	1.83	1.32
6	0.13	1.99	0.16	2.63	1.52	0.65
7	0.16	0.24	0.13	2.4	1.54	2.34
8	0.12	1.23	0.09	1.93	1.68	0.97
9	0.10	2.88	0.07	1.35	1.97	0.75
10	0.09	1.23	0.06	1.63	1.17	2.31
11	0.06	1.15	0.04	0.97	2.98	0.80
12(S)	0	0	0	0	0	0
13	0.07	0.24	0.09	1.31	0.77	2.20
14	0.09	0.63	0.13	1.55	2.81	1.92
15	0.09	1.69	0.12	1.23	1.02	0.58
16	0.11	0.56	0.14	1.53	1.93	1.76
17	0.15	0.58	0.16	0.84	2.68	1.26
18	0.17	0.84	0.17	2.89	0.76	2.39
19	0.19	0.76	0.19	1.68	2.56	1.93
20	0.14	0.55	0.18	1.66	1.33	1.36

RRT represents relative retention time; RPA represents relative peak area.

**Table 5:** The effects of different spectrum pretreated methods on the performance index.

Spectral preprocessing method	Performance index (PI)
MSC + spectrum + Ns^1^	93.2 ± 0.01
MSC + spectrum + S-G	93.2 ± 0.02
MSC + FD + Ns	94.6 ± 0.04
MSC + FD + S-G	94.5 ± 0.03
MSC + FD + Nd	95.0 ± 0.02
MSC + SD + Ns	94.8 ± 0.02
MSC + SD + S-G	95.3 ± 0.04
MSC + SD + Nd	94.6 ± 0.02

^1^NS, no smoothing.

**Table 6:** The average distances of samples within the same class and different class centers.

Samples in different classes	Centers of different classes			
	Henan	Shandong	Anhui	Sichuan
Henan	0.9859 ± 0.012	1.8614 ± 0.010	2.4138 ± 0.091	4.0963 ± 0.011
Shandong	1.9862 ± 0.015	0.8769 ± 0.011	2.2174 ± 0.089	4.1573 ± 0.098
Anhui	2.5375 ± 0.021	2.0167 ± 0.093	0.9788 ± 0.010	2.8943 ± 0.012
Sichuan	4.9736 ± 0.009	3.8794 ± 0.012	3.1027 ± 0.014	0.8796 ± 0.025

## Data Availability

The data used to support the findings of this study are available from the corresponding author upon request.

## Supplementary Materials

Supplementary Materials The chromatograms of *Salvia miltiorrhiza* Bunge from different places and near-infrared (NIR) spectroscopy data of *Salvia miltiorrhiza* Bunge from different places.

## References

[1] Nagulapalli Venkata K. C., Swaroop A., Bagchi D., Bishayee A. (2017). A small plant with big benefits: fenugreek (*Trigonella foenum-graecum* Linn.) for disease prevention and health promotion. *Molecular Nutrition & Food Research*.

[2] Singab A.-N. B., El-Hefnawy H. M., Esmat A., Gad H. A., Nazeam J. A. (2015). A systemic review on aloe arborescens pharmacological profile: biological activities and pilot clinical trials. *Phytotherapy Research*.

[3] Farnsworth N. R., Akerele O., Bingel A. S. (1987). Medicinal plants in therapy. *Journal of Ethnopharmacology*.

[4] Walzer S. M., Weinmann D., Toegel S. (2015). Medical plant extracts for treating knee osteoarthritis: a snapshot of recent clinical trials and their biological background. *Current Rheumatology Reports*.

[5] Ayrle H., Mevissen M., Kaske M. (2016). Medicinal plants—prophylactic and therapeutic options for gastrointestinal and respiratory diseases in calves and piglets? A systematic review. *BMC Veterinary Research*.

[6] Tang T. Y., Li F.-z., Afseth J. (2014). Review of the regulations for clinical research in herbal medicines in USA. *Chinese Journal of Integrative Medicine*.

[7] Lv C., Wu X., Wang X. (2017). The gene expression profiles in response to 102 traditional Chinese medicine (TCM) components: a general template for research on TCMs. *Scientific Reports*.

[8] Li S., Zhang B. (2013). Traditional Chinese medicine network pharmacology: theory, methodology and application. *Chinese Journal of Natural Medicines*.

[9] Suo T., Liu J., Chen X. (2017). Combining chemical profiling and network analysis to investigate the pharmacology of complex prescriptions in traditional Chinese medicine. *Scientific Reports*.

[10] Sheridan H., Krenn L., Jiang R. (2012). The potential of metabolic fingerprinting as a tool for the modernisation of TCM preparations. *Journal of Ethnopharmacology*.

[11] Yang D.-Z., An Y.-Q., Jiang X.-L. (2011). Development of a novel method combining HPLC fingerprint and multi-ingredients quantitative analysis for quality evaluation of traditional Chinese medicine preparation. *Talanta*.

[12] Chen P., Luthria D., Harrington P. B., Harnly J. M. (2011). Discrimination among Panax species using spectral fingerprinting. *Journal of AOAC International*.

[13] Buratti S., Sinelli N., Bertone E., Venturello A., Casiraghi E., Geobaldo F. (2014). Discrimination between washed *Arabica*, natural *Arabica* and *Robusta* coffees discrimination using NIR spectroscopy, electronic nose and electronic tongue analysis. *Journal of the Science of Food and Agriculture*.

[14] Casale M., Oliveri P., Armanino C., Lanteri S., Forina M. (2010). NIR and UV-vis spectroscopy, artificial nose and tongue: comparison of four fingerprinting techniques for the characterisation of Italian red wines. *Analytica Chimica Acta*.

[15] Fu H., Fan Y., Zhang X. (2015). Rapid discrimination for traditional complex herbal medicines from different parts, collection time, and origins using high-performance liquid chromatography and near-infrared spectral fingerprints with aid of pattern recognition methods. *Journal of Analytical Methods in Chemistry*.

[16] Lu J., Xiang B., Liu H. (2008). Application of two-dimensional near-infrared correlation spectroscopy to the discrimination of Chinese herbal medicine of different geographic regions. *Spectrochimica Acta Part A: Molecular and Biomolecular Spectroscopy*.

[17] Zhao H., Guo B., Wei Y., Zhang B. (2014). Effects of grown origin, genotype, harvest year, and their interactions of wheat kernels on near infrared spectral fingerprints for geographical traceability. *Food Chemistry*.

[18] Zhu J., Fan X., Cheng Y. (2014). Chemometric analysis for identification of botanical raw materials for pharmaceutical use: a case study using Panax notoginseng. *PLoS One*.

[19] Guo Y., Li Y., Xue L. (2014). *Salvia miltiorrhiza*: an ancient Chinese herbal medicine as a source for anti-osteoporotic drugs. *Journal of Ethnopharmacology*.

[20] Ma P., Liu J., Zhang C., Liang Z. (2013). Regulation of water-soluble phenolic acid biosynthesis in *Salvia miltiorrhiza* Bunge. *Applied Biochemistry and Biotechnology*.

[21] Sung B., Chung H. S., Kim M. (2015). Cytotoxic effects of solvent-extracted active components of *Salvia miltiorrhiza* Bunge on human cancer cell lines. *Experimental and Therapeutic Medicine*.

[22] Tung N. H., Nakajima K., Uto T. (2017). Bioactive triterpenes from the root of *Salvia miltiorrhiza* Bunge. *Phytotherapy Research*.

[23] Wang Z., Ma S., Zhang Q. (2017). Matrix solid-phase dispersion coupled with high-performance liquid chromatography diode array detection for simultaneous determination of four lipophilic constituents from *Salvia miltiorrhiza* Bunge. *Journal of Chromatographic Science*.

[24] Hung Y.-C., Pan T.-L., Hu W.-L. (2016). Roles of reactive oxygen species in anticancer therapy with *Salvia miltiorrhiza* Bunge. *Oxidative Medicine and Cellular Longevity*.

[25] Jiang Y., Zhang L., Rupasinghe H. P. V. (2017). Antiproliferative effects of extracts from Salvia officinalis L. and Saliva miltiorrhiza Bunge on hepatocellular carcinoma cells. *Biomedicine & Pharmacotherapy*.

[26] Yuan T., Chen Y., Zhang H., Fang L., Du G. (2017). Salvianolic acid A, a component of *Salvia miltiorrhiza*, attenuates endothelial-mesenchymal transition of HPAECs induced by hypoxia. *The American Journal of Chinese Medicine*.

[27] Zhang Y., Xie Y., Liao X., Jia Q., Chai Y., Phytopharmacology (2017). A Chinese patent medicine *Salvia miltiorrhiza* depside salts for infusion combined with conventional treatment for patients with angina pectoris: a systematic review and meta-analysis of randomized controlled trials. *Phytomedicine*.

[28] Hu L., Ma S., Yin C. (2018). Discrimination of geographical origin and detection of adulteration of kudzu root by fluorescence spectroscopy coupled with multi-way pattern recognition. *Spectrochimica Acta Part A: Molecular and Biomolecular Spectroscopy*.

[29] Li C., Yang S.-C., Guo Q.-S., Zheng K.-Y., Wang P.-L., Meng Z.-G. (2016). Geographical traceability of Marsdenia tenacissima by Fourier transform infrared spectroscopy and chemometrics. *Spectrochimica Acta Part A: Molecular and Biomolecular Spectroscopy*.

[30] Tang J. F., Li W. X., Zhang F. (2017). Discrimination of Radix Polygoni Multiflori from different geographical areas by UPLC-QTOF/MS combined with chemometrics. *Chinese Medicine*.

[31] Zhao Y., Ma X., Fan L. (2017). Discrimination of geographical origin of cultivated Polygala tenuifolia based on multi-element fingerprinting by inductively coupled plasma mass spectrometry. *Scientific Reports*.

