# Supplementary material for: Discrimination of Salvia miltiorrhiza Bunge from Different Geographical Locations Employing High-Performance Liquid Chromatography, Near-Infrared Fingerprinting Combined with Chemometrics
Source: J Anal Methods Chem. 2020 Feb 10;2020:8367619. doi: 10.1155/2020/8367619 (PMC7035550; doi:10.1155/2020/8367619)
Supplement: Supplementary Materials — The chromatograms of Salvia miltiorrhiza Bunge from different places and near-infrared (NIR) spectroscopy data of Salvia miltiorrhiza Bunge from different places. [file 8367619.f1.zip › 8367619.f1/1.docx]

混标

| # | 时间 | 峰面积 | 峰高 | 峰宽 | 对称因子 | 峰面积 % | 类型 |
| --- | --- | --- | --- | --- | --- | --- | --- |
| 1 | 8.614 | 122.6 | 13.7 | 0.1365 | 0.961 | 2.190 | BB |
| 2 | 11.586 | 79.2 | 8.3 | 0.1474 | 0.92 | 1.414 | BB |
| 3 | 13.473 | 48.2 | 5.3 | 0.1424 | 0.898 | 0.861 | BB |
| 4 | 30.547 | 767.3 | 44.1 | 0.2668 | 0.919 | 13.703 | BB |
| 5 | 37.36 | 3726.2 | 158.1 | 0.345 | 0.399 | 66.546 | BB |
| 6 | 69.473 | 243.5 | 17.4 | 0.2151 | 0.858 | 4.348 | BB |
| 7 | 74.841 | 237.5 | 17.8 | 0.208 | 0.939 | 4.241 | BB |
| 8 | 75.513 | 77 | 5.9 | 0.2038 | 0.942 | 1.375 | BB |
| 9 | 80.746 | 298 | 22.4 | 0.2074 | 0.91 | 5.322 | BB |

21号

| # | 时间 | 峰面积 | 峰高 | 峰宽 | 对称因子 | 峰面积 % | 类型 |
| --- | --- | --- | --- | --- | --- | --- | --- |
| 1 | 13.416 | 15.3 | 1.8 | 0.1363 | 1.133 | 0.237 | BB |
| 2 | 30.428 | 584.6 | 34.5 | 0.2619 | 0.932 | 9.038 | BB |
| 3 | 37.122 | 4531.1 | 208.4 | 0.3277 | 0.384 | 70.054 | BB |
| 4 | 69.444 | 67.2 | 4.7 | 0.2202 | 1.008 | 1.039 | BB |
| 5 | 74.817 | 389.1 | 28.4 | 0.2118 | 0.92 | 6.015 | BV |
| 6 | 75.488 | 166.5 | 10.3 | 0.2373 | 0.843 | 2.574 | VB |
| 7 | 80.712 | 714.3 | 53.6 | 0.2075 | 0.912 | 11.044 | BB |

22

| # | 时间 | 峰面积 | 峰高 | 峰宽 | 对称因子 | 峰面积 % | 类型 |
| --- | --- | --- | --- | --- | --- | --- | --- |
| 1 | 13.381 | 11.7 | 1.3 | 0.1397 | 1.231 | 0.199 | BB |
| 2 | 30.454 | 493.6 | 29.1 | 0.2621 | 0.92 | 8.423 | BB |
| 3 | 37.223 | 3562.3 | 172.3 | 0.3152 | 0.428 | 60.784 | BB |
| 4 | 69.444 | 76 | 5.2 | 0.2198 | 0.978 | 1.297 | BB |
| 5 | 74.805 | 483.2 | 35.2 | 0.2122 | 0.921 | 8.244 | BV |
| 6 | 75.479 | 256.5 | 15.6 | 0.2418 | 0.847 | 4.377 | VB |
| 7 | 80.695 | 977.3 | 73.5 | 0.2052 | 0.914 | 16.676 | BB |

23号样

| # | 时间 | 峰面积 | 峰高 | 峰宽 | 对称因子 | 峰面积 % | 类型 |
| --- | --- | --- | --- | --- | --- | --- | --- |
| 1 | 13.388 | 13.9 | 1.5 | 0.1388 | 1.22 | 0.243 | BB |
| 2 | 30.422 | 431.5 | 25.3 | 0.2627 | 0.922 | 7.533 | BB |
| 3 | 37.207 | 3499 | 170.7 | 0.3132 | 0.433 | 61.089 | BB |
| 4 | 69.435 | 102.8 | 7.1 | 0.222 | 0.939 | 1.795 | BB |
| 5 | 74.797 | 627.7 | 45.9 | 0.2116 | 0.926 | 10.960 | BV |
| 6 | 75.467 | 238.7 | 14.5 | 0.242 | 0.854 | 4.168 | VB |
| 7 | 80.68 | 814 | 61.4 | 0.2047 | 0.915 | 14.211 | BB |

24

| # | 时间 | 峰面积 | 峰高 | 峰宽 | 对称因子 | 峰面积 % | 类型 |
| --- | --- | --- | --- | --- | --- | --- | --- |
| 1 | 11.522 | 10.5 | 1 | 0.1509 | 1.191 | 0.170 | BB |
| 2 | 13.381 | 34.7 | 4.1 | 0.1337 | 1.027 | 0.560 | BB |
| 3 | 30.367 | 543 | 31.5 | 0.2649 | 0.932 | 8.761 | BB |
| 4 | 37.126 | 4292.5 | 195.5 | 0.3322 | 0.387 | 69.262 | BB |
| 5 | 69.365 | 109 | 7.1 | 0.2287 | 0.86 | 1.760 | BB |
| 6 | 74.744 | 442.7 | 32.2 | 0.2122 | 0.916 | 7.143 | BV |
| 7 | 75.419 | 169.3 | 10.6 | 0.2371 | 0.887 | 2.732 | VB |
| 8 | 80.64 | 595.7 | 45 | 0.2066 | 0.916 | 9.612 | BB |

25号

| # | 时间 | 峰面积 | 峰高 | 峰宽 | 对称因子 | 峰面积 % | 类型 |
| --- | --- | --- | --- | --- | --- | --- | --- |
| 1 | 13.368 | 13.8 | 1.6 | 0.1363 | 1.082 | 0.304 | BB |
| 2 | 30.396 | 501.1 | 29.7 | 0.2586 | 0.925 | 11.067 | BB |
| 3 | 37.164 | 3595.3 | 173.2 | 0.3182 | 0.424 | 79.402 | BB |
| 4 | 69.394 | 20 | 1.3 | 0.2332 | 1.295 | 0.441 | BB |
| 5 | 74.783 | 80.4 | 6 | 0.2095 | 0.935 | 1.776 | BB |
| 6 | 75.458 | 49.5 | 3.5 | 0.2151 | 1.031 | 1.093 | BB |
| 7 | 80.705 | 267.9 | 20 | 0.2081 | 0.912 | 5.917 | BB |

混标

| # | 时间 | 峰面积 | 峰高 | 峰宽 | 对称因子 | 峰面积 % | 类型 |
| --- | --- | --- | --- | --- | --- | --- | --- |
| 1 | 8.538 | 128.9 | 14.4 | 0.1383 | 0.961 | 2.194 | BB |
| 2 | 11.497 | 83.9 | 8.7 | 0.148 | 0.93 | 1.427 | BB |
| 3 | 13.371 | 50.9 | 5.5 | 0.144 | 0.894 | 0.865 | BB |
| 4 | 30.342 | 792.3 | 46 | 0.267 | 0.947 | 13.480 | BB |
| 5 | 37.167 | 3918.5 | 166.2 | 0.3471 | 0.389 | 66.665 | BB |
| 6 | 69.408 | 258 | 18.6 | 0.2134 | 0.861 | 4.390 | BB |
| 7 | 74.73 | 251.9 | 18.9 | 0.2074 | 0.938 | 4.286 | BB |
| 8 | 75.396 | 80.6 | 6.2 | 0.2006 | 0.946 | 1.371 | BB |
| 9 | 80.605 | 312.9 | 23.4 | 0.2057 | 0.91 | 5.323 | BB |

26号

| # | 时间 | 峰面积 | 峰高 | 峰宽 | 对称因子 | 峰面积 % | 类型 |
| --- | --- | --- | --- | --- | --- | --- | --- |
| 1 | 11.483 | 19.2 | 1.1 | 0.2305 | 2.188 | 0.378 | BB |
| 2 | 13.377 | 20.5 | 2.1 | 0.1453 | 0.866 | 0.404 | BB |
| 3 | 30.414 | 1378.7 | 82.3 | 0.2595 | 0.918 | 27.171 | BB |
| 4 | 37.247 | 2808.6 | 141.6 | 0.3054 | 0.474 | 55.350 | BB |
| 5 | 69.404 | 58.9 | 4.1 | 0.2169 | 1.036 | 1.160 | BB |
| 6 | 74.725 | 162.1 | 12.1 | 0.2087 | 0.938 | 3.194 | BB |
| 7 | 75.396 | 111 | 7.9 | 0.2144 | 0.997 | 2.187 | BB |
| 8 | 80.603 | 515.3 | 38.6 | 0.2077 | 0.909 | 10.155 | BB |

27号

| # | 时间 | 峰面积 | 峰高 | 峰宽 | 对称因子 | 峰面积 % | 类型 |
| --- | --- | --- | --- | --- | --- | --- | --- |
| 1 | 13.374 | 20.9 | 2.2 | 0.149 | 0.939 | 0.393 | BB |
| 2 | 30.335 | 1147.1 | 68 | 0.2608 | 0.918 | 21.581 | BB |
| 3 | 37.13 | 3196.7 | 156.7 | 0.312 | 0.445 | 60.140 | BB |
| 4 | 69.408 | 83.3 | 5.9 | 0.2171 | 1.001 | 1.567 | BB |
| 5 | 74.779 | 257.5 | 19 | 0.2099 | 0.933 | 4.844 | BB |
| 6 | 75.446 | 131.3 | 8.6 | 0.2278 | 0.815 | 2.470 | BB |
| 7 | 80.677 | 478.6 | 36.1 | 0.2045 | 0.912 | 9.004 | BB |

28

| # | 时间 | 峰面积 | 峰高 | 峰宽 | 对称因子 | 峰面积 % | 类型 |
| --- | --- | --- | --- | --- | --- | --- | --- |
| 1 | 13.358 | 30.9 | 3.4 | 0.1414 | 0.954 | 0.431 | BB |
| 2 | 30.379 | 1465.2 | 87 | 0.2606 | 0.922 | 20.411 | BB |
| 3 | 37.119 | 4871 | 217.9 | 0.3327 | 0.365 | 67.854 | BV R |
| 4 | 69.457 | 102 | 7.1 | 0.2192 | 1.016 | 1.420 | BB |
| 5 | 74.84 | 312.5 | 23.1 | 0.2099 | 0.935 | 4.353 | BB |
| 6 | 75.504 | 87.4 | 6.2 | 0.2168 | 0.974 | 1.217 | BB |
| 7 | 80.743 | 309.6 | 23.2 | 0.2056 | 0.915 | 4.313 | BB |

29号

| # | 时间 | 峰面积 | 峰高 | 峰宽 | 对称因子 | 峰面积 % | 类型 |
| --- | --- | --- | --- | --- | --- | --- | --- |
| 1 | 11.518 | 14.7 | 1.4 | 0.1544 | 1.14 | 0.258 | BB |
| 2 | 13.36 | 21.2 | 2.5 | 0.1346 | 1.005 | 0.373 | BB |
| 3 | 30.399 | 699.5 | 41.5 | 0.2608 | 0.935 | 12.286 | BB |
| 4 | 37.175 | 3566.7 | 173.8 | 0.3114 | 0.425 | 62.645 | BB |
| 5 | 69.434 | 87.3 | 5.6 | 0.233 | 1.056 | 1.534 | BB |
| 6 | 74.813 | 439.7 | 32.1 | 0.212 | 0.919 | 7.723 | BV |
| 7 | 75.474 | 210.9 | 13.6 | 0.2314 | 0.944 | 3.704 | VB |
| 8 | 80.702 | 653.4 | 49.3 | 0.2045 | 0.914 | 11.477 | BB |

30号样

| # | 时间 | 峰面积 | 峰高 | 峰宽 | 对称因子 | 峰面积 % | 类型 |
| --- | --- | --- | --- | --- | --- | --- | --- |
| 1 | 11.509 | 15.9 | 1.5 | 0.1559 | 1.09 | 0.276 | BB |
| 2 | 13.354 | 20.1 | 2.3 | 0.135 | 0.983 | 0.350 | BB |
| 3 | 30.426 | 869.1 | 51.4 | 0.2614 | 0.928 | 15.109 | BB |
| 4 | 37.216 | 3728.7 | 179.2 | 0.3147 | 0.415 | 64.822 | BB |
| 5 | 69.455 | 47.1 | 3.1 | 0.2239 | 1.108 | 0.818 | BB |
| 6 | 74.838 | 271.7 | 20.1 | 0.2097 | 0.934 | 4.724 | BB |
| 7 | 75.502 | 163.1 | 11 | 0.2248 | 0.932 | 2.836 | BB |
| 8 | 80.741 | 636.5 | 48.1 | 0.2044 | 0.909 | 11.066 | BB |

混标

| # | 时间 | 峰面积 | 峰高 | 峰宽 | 对称因子 | 峰面积 % | 类型 |
| --- | --- | --- | --- | --- | --- | --- | --- |
| 1 | 8.561 | 140.6 | 15.9 | 0.1372 | 0.959 | 2.225 | BB |
| 2 | 11.498 | 93.1 | 9.7 | 0.1478 | 0.924 | 1.473 | BB |
| 3 | 13.36 | 65.8 | 6.1 | 0.1626 | 1.196 | 1.042 | VB R |
| 4 | 30.391 | 881.9 | 50.1 | 0.2712 | 0.915 | 13.958 | BB |
| 5 | 37.211 | 4217.1 | 175.8 | 0.3518 | 0.376 | 66.743 | BB |
| 6 | 69.462 | 277 | 19.9 | 0.2125 | 0.873 | 4.384 | BB |
| 7 | 74.84 | 228.3 | 17.3 | 0.2057 | 0.941 | 3.614 | BB |
| 8 | 75.496 | 87.8 | 6.8 | 0.2016 | 0.945 | 1.389 | BB |
| 9 | 80.669 | 326.8 | 24.6 | 0.205 | 0.906 | 5.172 | BB |

31号

| # | 时间 | 峰面积 | 峰高 | 峰宽 | 对称因子 | 峰面积 % | 类型 |
| --- | --- | --- | --- | --- | --- | --- | --- |
| 1 | 8.305 | 16.5 | 1.8 | 0.1415 | 0.985 | 0.148 | VV |
| 2 | 11.52 | 16.9 | 1.7 | 0.1508 | 1.204 | 0.152 | BB |
| 3 | 13.39 | 25.7 | 3 | 0.1345 | 0.987 | 0.230 | BB |
| 4 | 30.408 | 2312.5 | 138.2 | 0.2592 | 0.904 | 20.751 | BB |
| 5 | 37.1 | 5955.1 | 246.6 | 0.3598 | 0.328 | 53.437 | BB |
| 6 | 69.456 | 201.7 | 13.5 | 0.2241 | 0.906 | 1.810 | BB |
| 7 | 74.836 | 1306.3 | 95.6 | 0.2114 | 0.916 | 11.721 | BV |
| 8 | 75.502 | 292.6 | 17.4 | 0.2441 | 0.778 | 2.626 | VB |
| 9 | 80.721 | 1016.8 | 76.8 | 0.2065 | 0.91 | 9.124 | BB |

32

| # | 时间 | 峰面积 | 峰高 | 峰宽 | 对称因子 | 峰面积 % | 类型 |
| --- | --- | --- | --- | --- | --- | --- | --- |
| 1 | 13.379 | 25.3 | 2.9 | 0.1353 | 1.005 | 0.197 | BB |
| 2 | 30.351 | 3032.1 | 180.6 | 0.2598 | 0.893 | 23.542 | BB |
| 3 | 37.017 | 7566.2 | 304.7 | 0.3697 | 0.304 | 58.746 | BB |
| 4 | 69.455 | 134.6 | 9.3 | 0.2213 | 0.988 | 1.045 | BB |
| 5 | 74.813 | 856.3 | 62.6 | 0.2115 | 0.915 | 6.648 | BV |
| 6 | 75.479 | 302.9 | 18.7 | 0.2391 | 0.777 | 2.352 | VB |
| 7 | 80.716 | 962.2 | 71.9 | 0.2081 | 0.913 | 7.471 | BB |

33

| # | 时间 | 峰面积 | 峰高 | 峰宽 | 对称因子 | 峰面积 % | 类型 |
| --- | --- | --- | --- | --- | --- | --- | --- |
| 1 | 11.522 | 8.7 | 1.1 | 0.1295 | 0.736 | 0.113 | BB |
| 2 | 13.399 | 27.1 | 3.1 | 0.1345 | 0.974 | 0.354 | BB |
| 3 | 30.42 | 1683.3 | 101 | 0.2585 | 0.92 | 22.002 | BB |
| 4 | 37.159 | 4815.2 | 225.7 | 0.3229 | 0.38 | 62.937 | BB |
| 5 | 69.411 | 70.5 | 4.8 | 0.2227 | 1.072 | 0.922 | BB |
| 6 | 74.789 | 422 | 31.1 | 0.2101 | 0.928 | 5.516 | BB |
| 7 | 75.461 | 144.5 | 8.8 | 0.2407 | 0.728 | 1.889 | BB |
| 8 | 80.689 | 479.5 | 35.8 | 0.2065 | 0.91 | 6.267 | BB |

1

| # | 时间 | 峰面积 | 峰高 | 峰宽 | 对称因子 | 峰面积 % | 类型 |
| --- | --- | --- | --- | --- | --- | --- | --- |
| 1 | 13.382 | 18.9 | 2.2 | 0.1355 | 1.062 | 0.348 | BB |
| 2 | 30.431 | 318.1 | 18.6 | 0.2633 | 0.921 | 5.866 | BB |
| 3 | 37.131 | 4833.3 | 221.4 | 0.3307 | 0.374 | 89.144 | BB |
| 4 | 69.424 | 22.7 | 1.5 | 0.2286 | 1.054 | 0.419 | BB |
| 5 | 74.795 | 65.8 | 4.9 | 0.2088 | 0.937 | 1.213 | BB |
| 6 | 75.46 | 14.7 | 1 | 0.212 | 1.139 | 0.271 | BB |
| 7 | 80.687 | 148.5 | 11.1 | 0.2059 | 0.909 | 2.738 | BB |

2

| # | 时间 | 峰面积 | 峰高 | 峰宽 | 对称因子 | 峰面积 % | 类型 |
| --- | --- | --- | --- | --- | --- | --- | --- |
| 1 | 13.379 | 13.9 | 1.6 | 0.1329 | 1.081 | 0.373 | BB |
| 2 | 30.351 | 392.5 | 23.2 | 0.2613 | 0.928 | 10.553 | BB |
| 3 | 37.169 | 3216.3 | 160 | 0.3086 | 0.45 | 86.472 | BB |
| 4 | 74.793 | 30.6 | 2.3 | 0.2056 | 0.96 | 0.823 | BB |
| 5 | 80.68 | 66.2 | 5 | 0.2077 | 0.908 | 1.779 | BB |

3

| # | 时间 | 峰面积 | 峰高 | 峰宽 | 对称因子 | 峰面积 % | 类型 |
| --- | --- | --- | --- | --- | --- | --- | --- |
| 1 | 13.364 | 13.9 | 1.7 | 0.1303 | 1.064 | 0.332 | BB |
| 2 | 30.392 | 551.4 | 32.7 | 0.2608 | 0.926 | 13.148 | BB |
| 3 | 37.208 | 3214.2 | 159.5 | 0.3091 | 0.448 | 76.636 | BB |
| 4 | 69.418 | 22.5 | 1.5 | 0.2234 | 1.037 | 0.536 | BB |
| 5 | 74.785 | 87.2 | 6.5 | 0.2097 | 0.95 | 2.078 | BB |
| 6 | 75.452 | 40.2 | 2.9 | 0.2115 | 1.029 | 0.959 | BB |
| 7 | 80.678 | 264.7 | 19.9 | 0.2072 | 0.911 | 6.312 | BB |

混标

| # | 时间 | 峰面积 | 峰高 | 峰宽 | 对称因子 | 峰面积 % | 类型 |
| --- | --- | --- | --- | --- | --- | --- | --- |
| 1 | 8.552 | 151.8 | 16.7 | 0.1402 | 0.99 | 2.238 | BB |
| 2 | 11.504 | 100 | 10.4 | 0.1504 | 0.931 | 1.474 | BB |
| 3 | 13.357 | 70.9 | 6.5 | 0.164 | 1.204 | 1.046 | VB R |
| 4 | 30.33 | 951.5 | 53.5 | 0.2733 | 0.918 | 14.029 | BB |
| 5 | 37.155 | 4535.3 | 185.2 | 0.3576 | 0.368 | 66.870 | BB |
| 6 | 69.411 | 296.2 | 21.3 | 0.2122 | 0.873 | 4.368 | BB |
| 7 | 74.781 | 235.4 | 17.7 | 0.2069 | 0.946 | 3.471 | BB |
| 8 | 75.451 | 94.1 | 7.2 | 0.2047 | 0.957 | 1.388 | BB |
| 9 | 80.684 | 347.1 | 26 | 0.2056 | 0.908 | 5.118 | BB |

4

| # | 时间 | 峰面积 | 峰高 | 峰宽 | 对称因子 | 峰面积 % | 类型 |
| --- | --- | --- | --- | --- | --- | --- | --- |
| 1 | 13.362 | 29.4 | 3.4 | 0.1335 | 0.983 | 0.500 | BB |
| 2 | 30.349 | 903.3 | 53.6 | 0.2606 | 0.927 | 15.395 | BB |
| 3 | 37.169 | 3299.3 | 162.6 | 0.3087 | 0.445 | 56.231 | BB |
| 4 | 69.412 | 169.5 | 12.3 | 0.2108 | 0.922 | 2.888 | BB |
| 5 | 74.792 | 816.5 | 60.1 | 0.2106 | 0.924 | 13.916 | BB |
| 6 | 75.463 | 118.9 | 7.5 | 0.2352 | 0.806 | 2.027 | BB |
| 7 | 80.694 | 530.6 | 39.9 | 0.207 | 0.91 | 9.043 | BB |

5

| # | 时间 | 峰面积 | 峰高 | 峰宽 | 对称因子 | 峰面积 % | 类型 |
| --- | --- | --- | --- | --- | --- | --- | --- |
| 1 | 13.367 | 22.2 | 2.6 | 0.134 | 0.963 | 0.377 | BB |
| 2 | 30.406 | 1337.8 | 79.2 | 0.261 | 0.918 | 22.712 | BB |
| 3 | 37.188 | 3775.1 | 179.4 | 0.3175 | 0.416 | 64.092 | BB |
| 4 | 69.417 | 69.7 | 5 | 0.2132 | 0.949 | 1.183 | BB |
| 5 | 74.804 | 315.6 | 23.4 | 0.209 | 0.923 | 5.359 | BB |
| 6 | 75.464 | 57.9 | 4 | 0.216 | 0.979 | 0.983 | BB |
| 7 | 80.661 | 311.8 | 23.5 | 0.2048 | 0.907 | 5.294 | BB |

6

| # | 时间 | 峰面积 | 峰高 | 峰宽 | 对称因子 | 峰面积 % | 类型 |
| --- | --- | --- | --- | --- | --- | --- | --- |
| 1 | 13.381 | 15.4 | 1.8 | 0.1313 | 0.931 | 0.337 | BB |
| 2 | 30.384 | 1182.6 | 70.8 | 0.2588 | 0.92 | 25.881 | BB |
| 3 | 37.214 | 2974.5 | 147.7 | 0.309 | 0.467 | 65.098 | BB |
| 4 | 69.427 | 27 | 1.9 | 0.2115 | 1.025 | 0.591 | BB |
| 5 | 74.817 | 141.9 | 10.5 | 0.21 | 0.931 | 3.106 | BB |
| 6 | 80.695 | 227.9 | 17.2 | 0.207 | 0.907 | 4.988 | BB |

7

| # | 时间 | 峰面积 | 峰高 | 峰宽 | 对称因子 | 峰面积 % | 类型 |
| --- | --- | --- | --- | --- | --- | --- | --- |
| 1 | 11.514 | 9.1 | 1.1 | 0.1328 | 0.829 | 0.196 | BB |
| 2 | 13.38 | 12.6 | 1.5 | 0.1326 | 1.052 | 0.271 | BB |
| 3 | 30.434 | 502.9 | 29.4 | 0.2617 | 0.919 | 10.792 | BB |
| 4 | 37.303 | 2777.1 | 140.1 | 0.3033 | 0.48 | 59.589 | BB |
| 5 | 69.364 | 92.2 | 6.3 | 0.2219 | 0.972 | 1.978 | BB |
| 6 | 74.705 | 523 | 38.1 | 0.2124 | 0.914 | 11.223 | BV |
| 7 | 75.367 | 159.1 | 9.4 | 0.2469 | 0.849 | 3.415 | VB |
| 8 | 80.593 | 584.3 | 43.6 | 0.2085 | 0.908 | 12.538 | BB |

8

| # | 时间 | 峰面积 | 峰高 | 峰宽 | 对称因子 | 峰面积 % | 类型 |
| --- | --- | --- | --- | --- | --- | --- | --- |
| 1 | 11.496 | 11.4 | 1.3 | 0.1348 | 0.862 | 0.237 | BB |
| 2 | 13.355 | 19.5 | 2.3 | 0.131 | 1.015 | 0.403 | BB |
| 3 | 30.341 | 919.6 | 54.4 | 0.2612 | 0.919 | 19.067 | BB |
| 4 | 37.179 | 3052.5 | 150.8 | 0.3101 | 0.465 | 63.288 | BB |
| 5 | 69.426 | 82.5 | 5.8 | 0.2161 | 0.971 | 1.710 | BB |
| 6 | 74.809 | 275.3 | 20.3 | 0.2104 | 0.93 | 5.709 | BB |
| 7 | 75.476 | 82 | 5.1 | 0.24 | 0.799 | 1.700 | BB |
| 8 | 80.737 | 380.3 | 28.3 | 0.2089 | 0.911 | 7.885 | BB |

9

| # | 时间 | 峰面积 | 峰高 | 峰宽 | 对称因子 | 峰面积 % | 类型 |
| --- | --- | --- | --- | --- | --- | --- | --- |
| 1 | 11.507 | 12.8 | 1.4 | 0.1372 | 0.832 | 0.255 | BB |
| 2 | 13.381 | 22.4 | 2.7 | 0.1321 | 0.997 | 0.445 | BB |
| 3 | 30.436 | 1166.2 | 69.9 | 0.2567 | 0.928 | 23.148 | BB |
| 4 | 37.266 | 3377 | 163.4 | 0.3152 | 0.437 | 67.030 | BB |
| 5 | 69.479 | 41.3 | 2.8 | 0.2185 | 1.038 | 0.819 | BB |
| 6 | 74.847 | 118.6 | 8.8 | 0.2094 | 0.934 | 2.355 | BB |
| 7 | 75.51 | 48.3 | 3.4 | 0.2172 | 1.006 | 0.959 | BB |
| 8 | 80.742 | 251.5 | 18.9 | 0.2053 | 0.909 | 4.991 | BB |

混标

| # | 时间 | 峰面积 | 峰高 | 峰宽 | 对称因子 | 峰面积 % | 类型 |
| --- | --- | --- | --- | --- | --- | --- | --- |
| 1 | 8.556 | 157.5 | 17.4 | 0.1394 | 0.966 | 2.255 | BB |
| 2 | 11.516 | 105.4 | 10.9 | 0.1511 | 0.923 | 1.508 | BB |
| 3 | 13.385 | 73.1 | 6.7 | 0.1633 | 1.201 | 1.047 | VB R |
| 4 | 30.385 | 992.5 | 56.2 | 0.272 | 0.925 | 14.207 | BB |
| 5 | 37.216 | 4692.5 | 193.9 | 0.3524 | 0.361 | 67.169 | BB |
| 6 | 69.459 | 307.7 | 22.1 | 0.2145 | 0.872 | 4.405 | BB |
| 7 | 74.828 | 212.7 | 16 | 0.2073 | 0.951 | 3.045 | BB |
| 8 | 75.49 | 97.7 | 7.4 | 0.203 | 0.959 | 1.399 | BB |
| 9 | 80.718 | 346.8 | 25.9 | 0.2083 | 0.905 | 4.965 | BB |

10

| # | 时间 | 峰面积 | 峰高 | 峰宽 | 对称因子 | 峰面积 % | 类型 |
| --- | --- | --- | --- | --- | --- | --- | --- |
| 1 | 13.38 | 35.9 | 4.2 | 0.1345 | 0.952 | 0.783 | BB |
| 2 | 30.438 | 403.8 | 24.3 | 0.2602 | 0.938 | 8.815 | BB |
| 3 | 37.178 | 3547.8 | 169 | 0.315 | 0.427 | 77.456 | BB |
| 4 | 69.33 | 74.2 | 5.3 | 0.2119 | 0.977 | 1.620 | BB |
| 5 | 74.65 | 303.2 | 22.5 | 0.2094 | 0.923 | 6.620 | BB |
| 6 | 75.302 | 33.8 | 2.3 | 0.221 | 0.908 | 0.738 | BB |
| 7 | 80.518 | 181.8 | 13.5 | 0.2075 | 0.906 | 3.969 | BB |

11

| # | 时间 | 峰面积 | 峰高 | 峰宽 | 对称因子 | 峰面积 % | 类型 |
| --- | --- | --- | --- | --- | --- | --- | --- |
| 1 | 13.346 | 30.9 | 3.6 | 0.1339 | 0.957 | 0.645 | BB |
| 2 | 30.15 | 443.4 | 26.6 | 0.2566 | 0.972 | 9.246 | BB |
| 3 | 36.827 | 3679.7 | 178.3 | 0.3127 | 0.428 | 76.737 | BB |
| 4 | 69.241 | 77.7 | 5.6 | 0.2134 | 0.954 | 1.621 | BB |
| 5 | 74.536 | 328.3 | 24.2 | 0.21 | 0.922 | 6.847 | BB |
| 6 | 75.189 | 31.2 | 2.1 | 0.2217 | 0.886 | 0.650 | BB |
| 7 | 80.43 | 204 | 15.2 | 0.2064 | 0.903 | 4.254 | BB |

12

| # | 时间 | 峰面积 | 峰高 | 峰宽 | 对称因子 | 峰面积 % | 类型 |
| --- | --- | --- | --- | --- | --- | --- | --- |
| 1 | 13.364 | 22.6 | 2.6 | 0.1344 | 0.944 | 0.443 | BB |
| 2 | 30.173 | 463.9 | 27.6 | 0.2584 | 0.966 | 9.077 | BB |
| 3 | 36.882 | 3448.6 | 164.5 | 0.3186 | 0.443 | 67.481 | BB |
| 4 | 69.359 | 94.7 | 6.6 | 0.2182 | 0.944 | 1.853 | BB |
| 5 | 74.702 | 618.6 | 45.2 | 0.2138 | 0.926 | 12.105 | BB |
| 6 | 75.37 | 72.4 | 4.6 | 0.2341 | 0.801 | 1.416 | BB |
| 7 | 80.566 | 389.7 | 29 | 0.209 | 0.905 | 7.625 | BB |

13

| # | 时间 | 峰面积 | 峰高 | 峰宽 | 对称因子 | 峰面积 % | 类型 |
| --- | --- | --- | --- | --- | --- | --- | --- |
| 1 | 13.376 | 16.5 | 2 | 0.1325 | 0.909 | 0.307 | BB |
| 2 | 30.355 | 670.1 | 39.6 | 0.2613 | 0.954 | 12.431 | BB |
| 3 | 37.099 | 3776.2 | 180.2 | 0.3164 | 0.425 | 70.049 | BB |
| 4 | 69.392 | 54.4 | 3.7 | 0.2223 | 0.965 | 1.008 | BB |
| 5 | 74.746 | 428.9 | 31.6 | 0.2103 | 0.929 | 7.956 | BB |
| 6 | 75.405 | 67.5 | 4.4 | 0.2288 | 0.79 | 1.252 | BB |
| 7 | 80.609 | 377.2 | 28.2 | 0.2061 | 0.914 | 6.998 | BB |

14

| # | 时间 | 峰面积 | 峰高 | 峰宽 | 对称因子 | 峰面积 % | 类型 |
| --- | --- | --- | --- | --- | --- | --- | --- |
| 1 | 13.365 | 26.3 | 3 | 0.1357 | 0.955 | 0.398 | BB |
| 2 | 30.319 | 606.5 | 35.8 | 0.2614 | 0.96 | 9.171 | BB |
| 3 | 37.034 | 4427.4 | 207.5 | 0.323 | 0.399 | 66.946 | BB |
| 4 | 69.379 | 124.3 | 8.2 | 0.229 | 0.869 | 1.880 | BB |
| 5 | 74.742 | 696.8 | 51.1 | 0.2111 | 0.927 | 10.536 | BB |
| 6 | 75.407 | 134.3 | 9.2 | 0.2212 | 0.81 | 2.031 | BB |
| 7 | 80.638 | 597.7 | 44.7 | 0.2059 | 0.908 | 9.037 | BB |

15

| # | 时间 | 峰面积 | 峰高 | 峰宽 | 对称因子 | 峰面积 % | 类型 |
| --- | --- | --- | --- | --- | --- | --- | --- |
| 1 | 13.378 | 36.8 | 4.2 | 0.1364 | 0.966 | 0.554 | BB |
| 2 | 30.347 | 522.8 | 30.8 | 0.2621 | 0.966 | 7.870 | BB |
| 3 | 37.033 | 4516.7 | 212.3 | 0.3222 | 0.396 | 68.000 | BB |
| 4 | 69.322 | 123.5 | 8.1 | 0.2309 | 0.884 | 1.859 | BB |
| 5 | 74.682 | 745.6 | 54.2 | 0.2126 | 0.923 | 11.226 | BB |
| 6 | 75.351 | 119.8 | 8 | 0.2273 | 0.835 | 1.803 | BB |
| 7 | 80.54 | 577.1 | 42.7 | 0.2075 | 0.903 | 8.688 | BB |

混标

| # | 时间 | 峰面积 | 峰高 | 峰宽 | 对称因子 | 峰面积 % | 类型 |
| --- | --- | --- | --- | --- | --- | --- | --- |
| 1 | 8.533 | 129.4 | 14.6 | 0.1354 | 0.949 | 2.176 | BB |
| 2 | 11.483 | 82.5 | 8.6 | 0.1494 | 0.922 | 1.387 | BB |
| 3 | 13.337 | 51.1 | 5.6 | 0.1414 | 0.895 | 0.859 | BB |
| 4 | 30.29 | 820.6 | 46.9 | 0.2702 | 0.924 | 13.799 | BB |
| 5 | 37.077 | 3973.4 | 169.5 | 0.3435 | 0.399 | 66.818 | BB |
| 6 | 69.318 | 255.8 | 18.1 | 0.2145 | 0.858 | 4.302 | BB |
| 7 | 74.671 | 251.2 | 18.7 | 0.2088 | 0.941 | 4.224 | BB |
| 8 | 75.338 | 77.2 | 5.9 | 0.2057 | 0.945 | 1.299 | BB |
| 9 | 80.571 | 305.5 | 22.6 | 0.2075 | 0.906 | 5.137 | BB |

16

| # | 时间 | 峰面积 | 峰高 | 峰宽 | 对称因子 | 峰面积 % | 类型 |
| --- | --- | --- | --- | --- | --- | --- | --- |
| 1 | 13.356 | 33.9 | 4.1 | 0.1291 | 0.969 | 0.437 | BB |
| 2 | 30.313 | 834.6 | 49.9 | 0.2589 | 0.944 | 10.752 | BB |
| 3 | 37.021 | 4113.6 | 197.7 | 0.3147 | 0.414 | 52.995 | BB |
| 4 | 69.344 | 433.9 | 30.8 | 0.2166 | 0.902 | 5.590 | BB |
| 5 | 74.723 | 1436.8 | 104.6 | 0.2123 | 0.923 | 18.510 | BB |
| 6 | 75.392 | 173.1 | 11.3 | 0.2291 | 0.819 | 2.230 | BB |
| 7 | 80.635 | 736.4 | 54.4 | 0.2099 | 0.902 | 9.487 | BB |

17

| # | 时间 | 峰面积 | 峰高 | 峰宽 | 对称因子 | 峰面积 % | 类型 |
| --- | --- | --- | --- | --- | --- | --- | --- |
| 1 | 13.355 | 29.5 | 3.4 | 0.1352 | 0.934 | 0.309 | BB |
| 2 | 30.281 | 1244.6 | 74.1 | 0.2598 | 0.928 | 13.042 | BB |
| 3 | 36.88 | 6661.2 | 287.8 | 0.3441 | 0.327 | 69.798 | BB |
| 4 | 69.263 | 216.4 | 15.1 | 0.2178 | 0.94 | 2.268 | BB |
| 5 | 74.604 | 749.1 | 54.7 | 0.2137 | 0.925 | 7.850 | BB |
| 6 | 75.271 | 116.1 | 7.7 | 0.2271 | 0.822 | 1.217 | BB |
| 7 | 80.552 | 526.5 | 39.2 | 0.2065 | 0.903 | 5.517 | BB |

18

| # | 时间 | 峰面积 | 峰高 | 峰宽 | 对称因子 | 峰面积 % | 类型 |
| --- | --- | --- | --- | --- | --- | --- | --- |
| 1 | 13.364 | 20.3 | 2.2 | 0.1398 | 1.072 | 0.245 | BB |
| 2 | 30.269 | 1104.1 | 65.6 | 0.2602 | 0.93 | 13.344 | BB |
| 3 | 36.901 | 5925.7 | 258.6 | 0.3413 | 0.342 | 71.616 | BB |
| 4 | 69.251 | 81 | 5.6 | 0.2217 | 1.003 | 0.979 | BB |
| 5 | 74.596 | 513.4 | 37.4 | 0.2121 | 0.921 | 6.205 | BB |
| 6 | 75.265 | 95.2 | 6 | 0.237 | 0.842 | 1.151 | BB |
| 7 | 80.557 | 534.5 | 39.7 | 0.209 | 0.901 | 6.460 | BB |

19

| # | 时间 | 峰面积 | 峰高 | 峰宽 | 对称因子 | 峰面积 % | 类型 |
| --- | --- | --- | --- | --- | --- | --- | --- |
| 1 | 13.367 | 19.3 | 2.1 | 0.1416 | 1.107 | 0.208 | BB |
| 2 | 30.347 | 1176.1 | 70.2 | 0.2593 | 0.936 | 12.677 | BB |
| 3 | 36.955 | 5978.2 | 267.1 | 0.3371 | 0.348 | 64.433 | BB |
| 4 | 69.375 | 163.5 | 11.6 | 0.2138 | 0.924 | 1.762 | BB |
| 5 | 74.714 | 1019 | 75.7 | 0.2091 | 0.922 | 10.983 | BB |
| 6 | 75.367 | 158.5 | 10.2 | 0.23 | 0.82 | 1.708 | BB |
| 7 | 80.615 | 763.4 | 56.5 | 0.2097 | 0.903 | 8.228 | BB |

20

| # | 时间 | 峰面积 | 峰高 | 峰宽 | 对称因子 | 峰面积 % | 类型 |
| --- | --- | --- | --- | --- | --- | --- | --- |
| 1 | 13.378 | 16.4 | 1.9 | 0.1339 | 1.075 | 0.244 | BB |
| 2 | 30.378 | 695 | 41.4 | 0.26 | 0.937 | 10.376 | BB |
| 3 | 37.065 | 4566.3 | 209.8 | 0.3299 | 0.393 | 68.167 | BB |
| 4 | 69.387 | 74.6 | 4.9 | 0.2298 | 1.045 | 1.114 | BB |
| 5 | 74.741 | 380.2 | 28.4 | 0.2064 | 0.934 | 5.676 | BB |
| 6 | 75.392 | 175.1 | 11.4 | 0.2298 | 0.831 | 2.615 | BB |
| 7 | 80.637 | 791 | 58.9 | 0.2088 | 0.902 | 11.808 | BB |

混标

| # | 时间 | 峰面积 | 峰高 | 峰宽 | 对称因子 | 峰面积 % | 类型 |
| --- | --- | --- | --- | --- | --- | --- | --- |
| 1 | 8.54 | 134.6 | 15 | 0.1365 | 0.962 | 2.209 | BB |
| 2 | 11.496 | 88 | 9.1 | 0.1484 | 0.923 | 1.443 | BB |
| 3 | 13.359 | 53 | 5.8 | 0.1435 | 0.894 | 0.870 | BB |
| 4 | 30.3 | 846.4 | 48.1 | 0.2712 | 0.923 | 13.886 | BB |
| 5 | 37.107 | 4058.4 | 171.1 | 0.3488 | 0.4 | 66.584 | BB |
| 6 | 69.361 | 272.1 | 19.4 | 0.2136 | 0.859 | 4.465 | BB |
| 7 | 74.716 | 247.6 | 18.7 | 0.2063 | 0.945 | 4.063 | BB |
| 8 | 75.37 | 80 | 6.1 | 0.2031 | 0.943 | 1.312 | BB |
| 9 | 80.628 | 315.1 | 23.4 | 0.2092 | 0.901 | 5.169 | BB |

38

| # | 时间 | 峰面积 | 峰高 | 峰宽 | 对称因子 | 峰面积 % | 类型 |
| --- | --- | --- | --- | --- | --- | --- | --- |
| 1 | 11.477 | 32.3 | 2.8 | 0.168 | 1.598 | 0.970 | BB |
| 2 | 13.337 | 190.3 | 18.4 | 0.1544 | 1.092 | 5.719 | BB |
| 3 | 30.192 | 1190.2 | 72.7 | 0.253 | 0.963 | 35.777 | BB |
| 4 | 37.004 | 1162.1 | 65.7 | 0.2744 | 0.678 | 34.934 | BB |
| 5 | 69.293 | 47.8 | 3.3 | 0.2201 | 1.093 | 1.438 | BB |
| 6 | 74.62 | 312.5 | 23 | 0.2103 | 0.917 | 9.394 | BB |
| 7 | 80.492 | 391.5 | 29.4 | 0.2072 | 0.9 | 11.769 | BB |

39

| # | 时间 | 峰面积 | 峰高 | 峰宽 | 对称因子 | 峰面积 % | 类型 |
| --- | --- | --- | --- | --- | --- | --- | --- |
| 1 | 13.336 | 128.2 | 12.7 | 0.1519 | 1.016 | 3.285 | BB |
| 2 | 30.184 | 1659.9 | 101.9 | 0.254 | 0.939 | 42.539 | BB |
| 3 | 37.003 | 958.3 | 54.5 | 0.2731 | 0.719 | 24.557 | BB |
| 4 | 69.264 | 45.7 | 3.1 | 0.2205 | 1.155 | 1.171 | BB |
| 5 | 74.602 | 373.9 | 27.7 | 0.2096 | 0.908 | 9.583 | BB |
| 6 | 75.26 | 53.4 | 3.7 | 0.2187 | 0.814 | 1.369 | BB |
| 7 | 80.474 | 682.8 | 50.9 | 0.2086 | 0.911 | 17.497 | BB |

40

| # | 时间 | 峰面积 | 峰高 | 峰宽 | 对称因子 | 峰面积 % | 类型 |
| --- | --- | --- | --- | --- | --- | --- | --- |
| 1 | 11.484 | 24.2 | 2.1 | 0.1676 | 1.618 | 0.666 | BB |
| 2 | 13.341 | 136.8 | 13.7 | 0.1507 | 0.995 | 3.767 | BB |
| 3 | 30.238 | 1505.2 | 91.6 | 0.2556 | 0.942 | 41.453 | BB |
| 4 | 37.083 | 899.4 | 51.8 | 0.2687 | 0.734 | 24.770 | BB |
| 5 | 69.259 | 37.7 | 2.5 | 0.2214 | 1.18 | 1.038 | BB |
| 6 | 74.587 | 314.2 | 23.4 | 0.2086 | 0.923 | 8.654 | BB |
| 7 | 75.237 | 48.4 | 3.3 | 0.2182 | 0.814 | 1.334 | BB |
| 8 | 80.451 | 665.2 | 49.2 | 0.2096 | 0.905 | 18.318 | BB |
